# Spatial and multidimensional visualization of Indonesia's village health statistics

**DOI:** 10.1186/1476-072X-7-30

**Published:** 2008-06-11

**Authors:** Bambang Parmanto, Maria V Paramita, Wayan Sugiantara, Gede Pramana, Matthew Scotch, Donald S Burke

**Affiliations:** 1 Health Information Management, University of Pittsburgh, 6051 Forbes Tower, Pittsburgh, Pennsylvania, USA; 2 Center for Medical Informatics, Yale University, 300 George St, Suite 501, New Haven, Connecticut, USA; 3 Graduate School of Public Health, University of Pittsburgh, Pittsburgh, Pennsylvania, USA

## Abstract

**Background:**

A community health assessment (CHA) is used to identify and address health issues in a given population. Effective CHA requires timely and comprehensive information from a wide variety of sources, such as: socio-economic data, disease surveillance, healthcare utilization, environmental data, and health resource allocation.

Indonesia is a developing country with 235 million inhabitants over 13,000 islands. There are significant barriers to conducting CHA in developing countries like Indonesia, such as the high cost of computing resources and the lack of computing skills necessary to support such an assessment.

At the University of Pittsburgh, we have developed the Spatial OLAP (On-Line Analytical Processing) Visualization and Analysis Tool (SOVAT) for performing CHA. SOVAT combines Geographic Information System (GIS) technology along with an advanced multidimensional data warehouse structure to facilitate analysis of large, disparate health, environmental, population, and spatial data.

The objective of this paper is to demonstrate the potential of SOVAT for facilitating CHA among developing countries by using health, population, healthcare resources, and spatial data from Indonesia for use in two CHA cases studies.

**Results:**

Bureau of Statistics administered data sets from the Indonesian Census, and the Indonesian village statistics, were used in the case studies. The data consisted of: healthcare resources (number of healthcare professionals and facilities), population (census), morbidity and mortality, and spatial (GIS-formatted) information.

The data was formatted, combined, and populated into SOVAT for CHA use. Case study 1 involves the distribution of healthcare professionals in Indonesia, while case study 2 involves malaria mortality. Screen shots are shown for both cases. The results for the CHA were retrieved in seconds and presented through the geospatial and numerical SOVAT interface.

**Conclusion:**

The case studies show the potential of spatial and multidimensional analysis using SOVAT for community health assessment in developing countries. Since SOVAT is based primarily on open-source components and can be deployed using small personal computers, it is cost-effective for developing countries. Also, combining the strength in analysis and the ease of use makes tools like SOVAT ideal for healthcare professionals without extensive computer skills.

## Background

Effective community health assessment (CHA) requires timely and comprehensive information from a wide variety of sources [1]. A CHA might be conducted in order to: identify or predict community problems; develop strategies to solve the problems; manage resources allocation; and, in turn, improve the quality of life in the community. Data sets used to conduct CHA usually come from several sources such as socio-economic data, disease surveillance, healthcare utilization, healthcare resources, environmental, and health resource allocation. By integrating these data sets, CHA can identify factors that affect the health of a population and determine the availability of resources within the community to adequately address these factors. Geographic Information System (GIS) is a technology to store, manipulate, analyze, and display geographically referenced information. Outside of healthcare, it has been shown to be valuable in a wide range of situations such as urban planning, environmental resource management, emergency planning, and transportation forecasting. CHA has also started to use GIS. A survey conducted by Canadian health professionals shows that 70% of respondents felt that community health decision making could be enhanced using a GIS application [2]. A previous study by the authors suggests that in the United States, GIS is used in conjunction with other software to analyze health and population data and perform numerical-spatial problem solving in CHA [3]. Numerical analysis only involves numerical data such as the calculation of a morbidity or mortality rate, while spatial analysis only involves spatial data such as geographical coordinates (such as latitude and longitude). The public health decision-making process can be considerably enhanced by the development of decision-support tools that allow spatial and multidimensional processing of information relatively quickly and easily. We have developed such an integrated tool called SOVAT (Spatial OLAP (On-Line Analytical Processing) Visualization and Analytical Tool) that allows public health professionals to conduct data linkage and perform quick multidimensional analysis visually [4]. In this paper, we present spatial and multidimensional visualization techniques that can be used to conduct CHA in different administrative levels using data sets that are routinely collected by Indonesia's Bureau of Statistics. The case presents the potentials and challenges of using a visual decision-support system like SOVAT in developing countries such as Indonesia.

Indonesia, with its 235 million inhabitants and more than 13,000 islands, is the world's 4th largest country (in terms of population). The use of a spatial decision-support system for CHA can greatly enhance the health assessment process. Until recently, most of the visible CHAs in Indonesia were conducted by international organizations. For example:

• An environmental assessment after the Aceh tsunami (December 2004) to identify the environmental and community issues related to the disaster and to prioritize reconstruction processes [5].

• An assessment of the poverty and damage to health facilities after the Yogyakarta earthquake (May 2006) to provide a reconstruction plan for the victims [6].

These examples suggest that the challenges in integrating and analyzing data from various sources require skills and resources that can only be assembled in the face of catastrophic events and by international organizations. The availability of a visual decision-support system can simplify the process of data integration and analysis and make the CHA process affordable to local and regional government offices in Indonesia.

## Results

The results presented in this paper are case studies of CHA in Indonesia by combining numerical and spatial data sets. The source of the numerical data is primarily from the Village Potential Statistics (PODES) of 2003, while the source of the spatial data is from the Indonesian Bureau of Statistics. There were a total of 60,000 villages in Indonesia in 2003. Data are collected from each individual village and then aggregated into a sub-district level. We integrated the numerical data with spatial data consisting of three levels of administrative boundaries: provinces, counties, and sub-districts. Administrative divisions in Indonesia consist of four levels. The country is divided into 33 provinces, and each province is sudivided into counties/regencies/city, which are further subdivided into subdistricts (kecamatan), and again subdivided into villages. Since subdistrict is usually the lowest unit for planning and assessment, we use the first three administrative levels. The result of the integration is a multidimensional database of 33 provinces, 445 counties, and 4,000 sub-districts.

The village statistics data sets contain indicators that are related to environmental and health conditions. The health-related variables include disease outbreak and mortality number, primary water sources, waste-treatment facilities, number of health facilities and medical staffs, natural disaster, and pollution.

There are two study cases presented in this paper. The first one is the distribution of physicians in Indonesia. The second one is the comparison of mortality numbers due to malaria diseases in rural and urban areas.

### Case Study 1: The distribution of healthcare professionals in Indonesia

Indonesia has an uneven distribution of population among its major islands. More than two-thirds of the country's population of 235 million is squeezed into the small Java island, just over 7% of its land mass. By contrast, the island of Papua in the easternmost part of the country represents 22% of the total land mass, yet has only 1% of the population. Like most countries, healthcare facilities and healthcare professionals tend to be concentrated in urban areas. Multidimensional analysis can provide information as to whether rural areas are indeed underserved by healthcare professionals and facilities. It can also identify which rural areas are in greatest need of healthcare services. The analysis of CHA can potentially be useful for planning and resource allocation. In this case study, healthcare service is represented by the number of physicians in the region.

To analyze the number of healthcare professionals, two measurements have been constructed in SOVAT: the number of healthcare professionals and the ratio of healthcare professionals/100,000 population. Healthcare professionals consist of physicians, nurses, and midwives. A dimension called healthcare professional is constructed in SOVAT with physician, nurse, and mid-wife as its members. Using this dimension, researchers can select which healthcare professionals he or she wants to analyze by choosing one of the members of the dimension.

**Figure 1:**
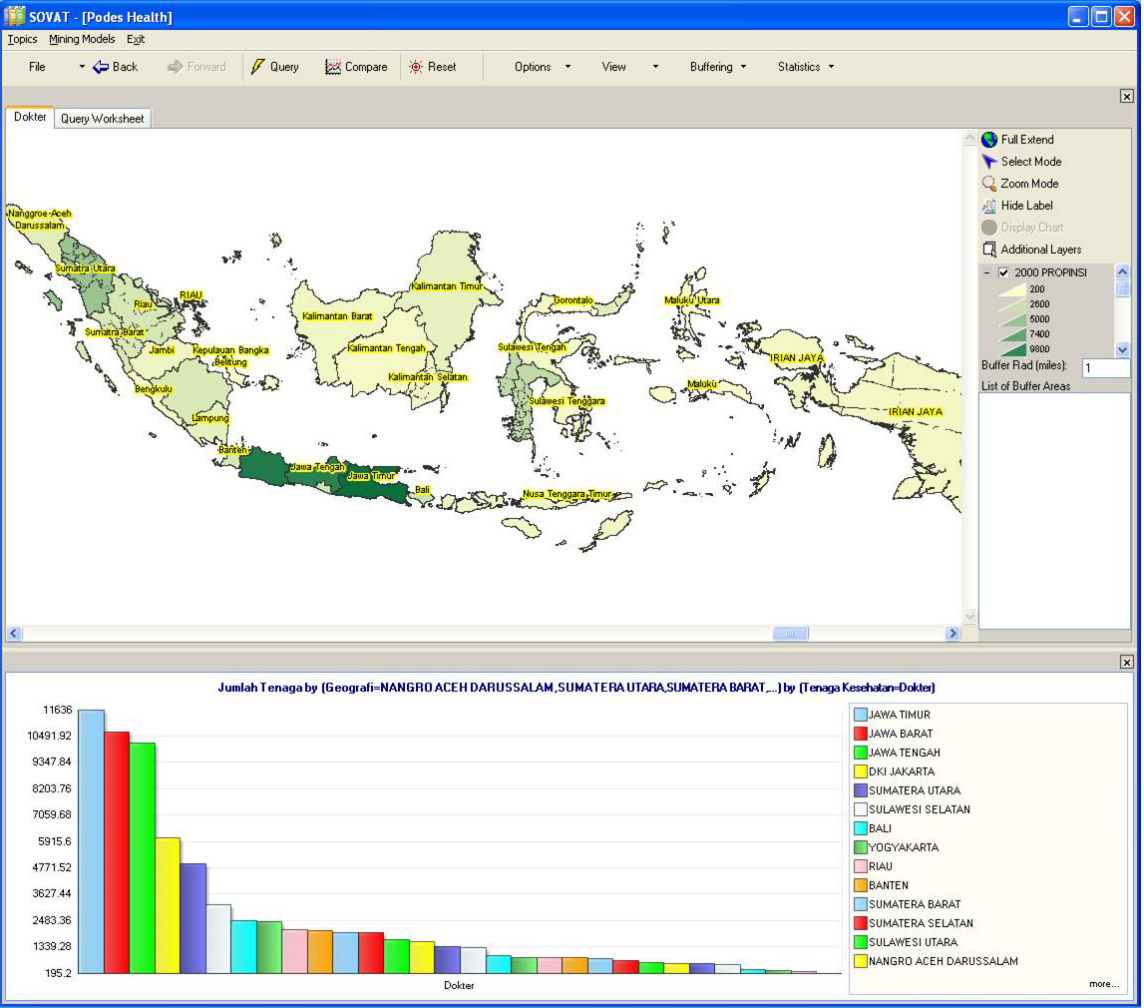
Number of Physicians in Each Province in Indonesia.

**Figure 2:**
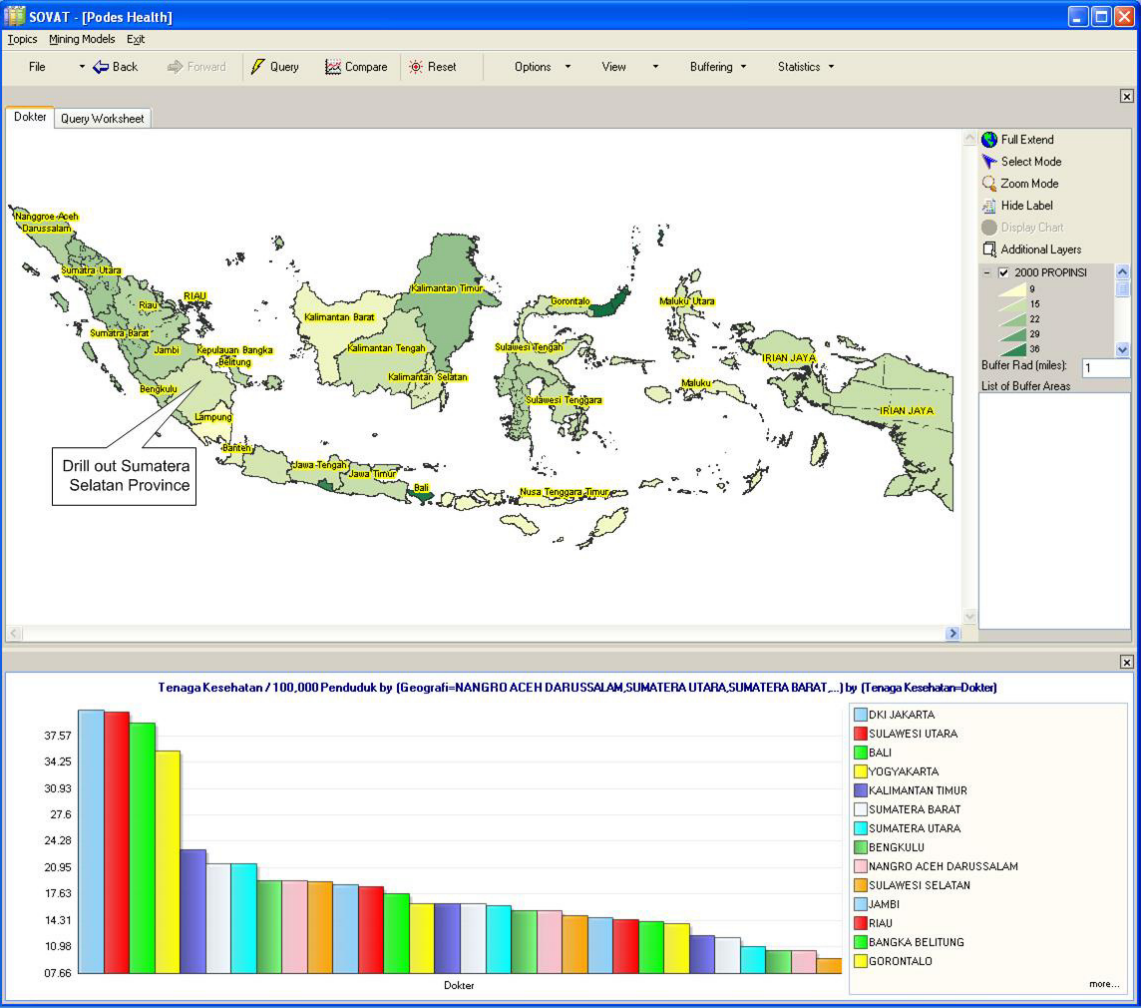
Number of Physicians/100,000 Populations in Each Province in Indonesia.

Figure 1 depicts the number of physicians in each province. As expected, the map shows that the concentration of physicians is in the island of Java (the area with the darkest color in the map). Since Java is by far also the most populated island, this map does not provide much information. The ratio of physicians/100,000 population represents a more accurate description of the distribution of physicians. This ratio is depicted in Figure 2, where it shows that the ratio of physicians to population in Java is not the highest one. This information is rather counter-intuitive, and can be very useful for decision making.

### Indonesia

**Figure 3:**
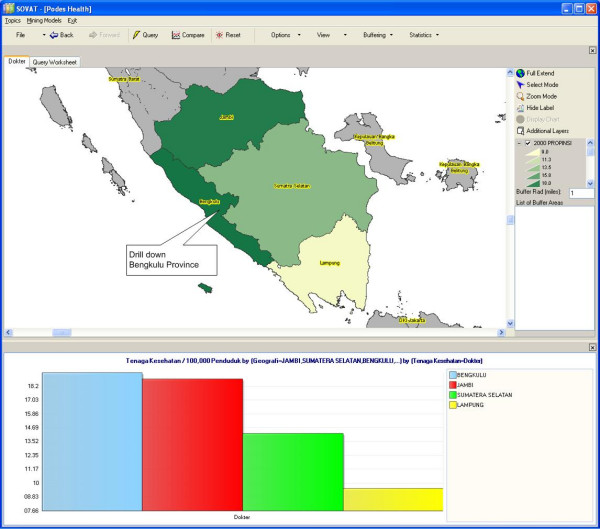
Number of Physicians/100,000 Populations in Southern part of Sumatra Island.

The analytical feature of SOVAT provides users with such functions as sorting the chart in ascending or descending order, and viewing only the top 5 or 10 results. Users can also focus on certain areas of the map to get more detailed information about the area. For example, suppose the user is interested in the province of South Sumatra and wants to compare it to neighboring provinces. To do that, the user can just select the South Sumatra province from the map and click *drill-out *from the pop-up menu. The result is shown as in Figure 3: the highest concentration of physicians in the southern part of Sumatra resides in the Bengkulu province.

**Figure 4:**
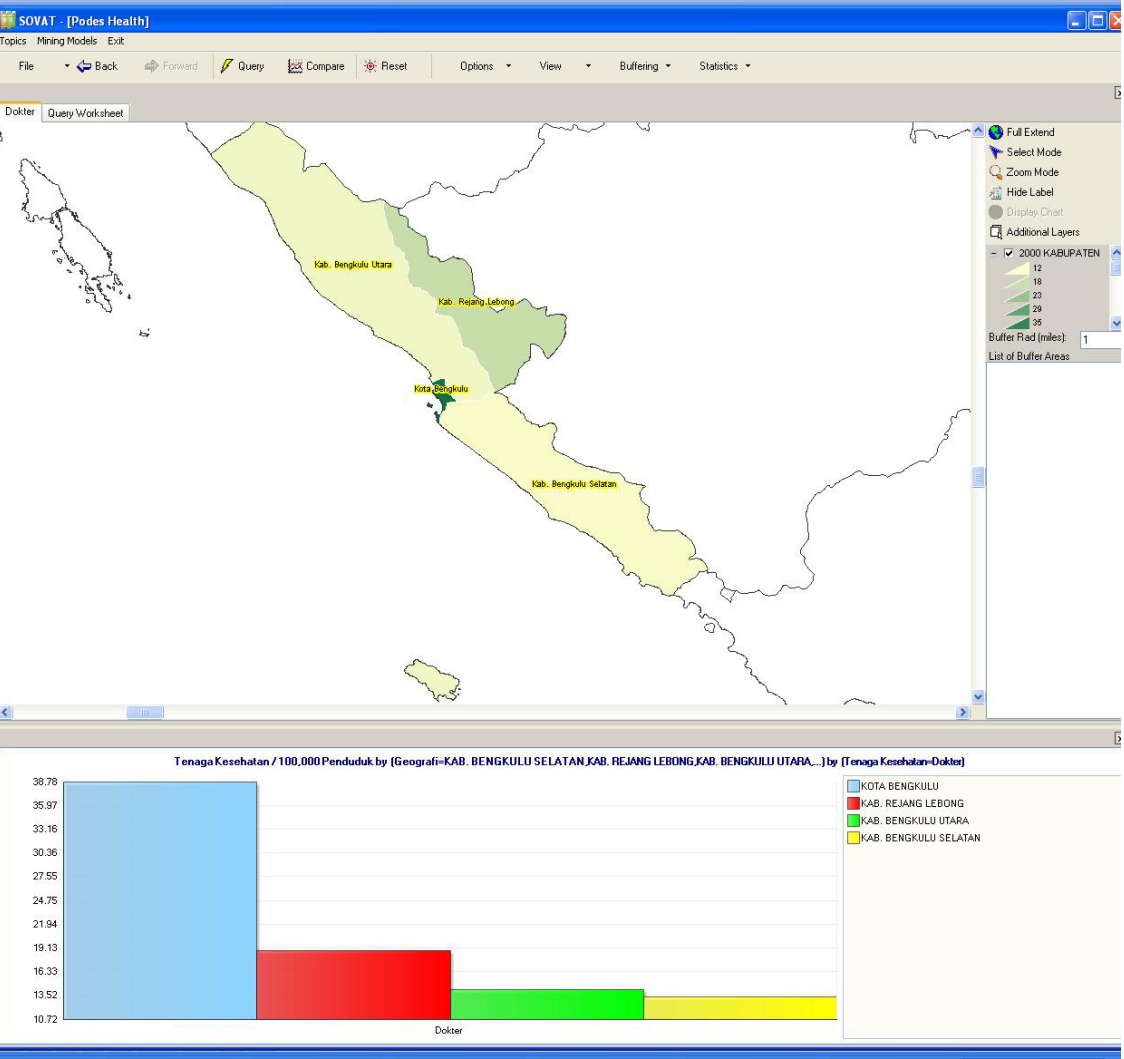
Number of Physicians/100,000 Populations in Bengkulu Province.

If the user wants to focus on all counties in one province, s/he can select the province and go to *drill-down*. Figure 4 shows the number of physicians in all counties in the Bengkulu province.

**Figure 5:**
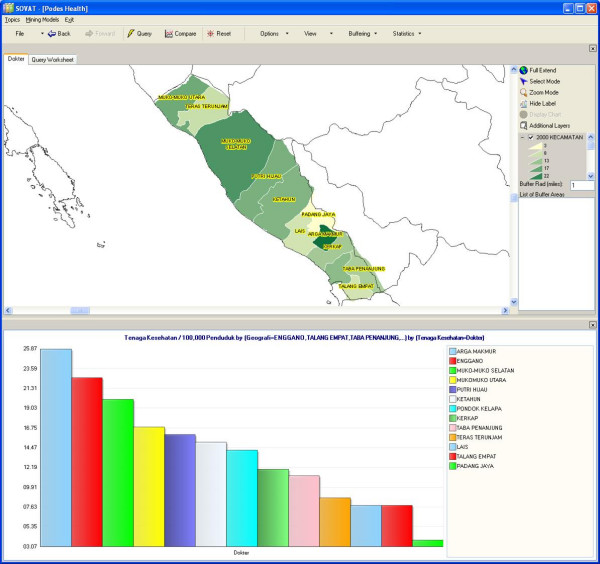
Number of Physicians/100,000 Populations in Bengkulu Utara.

Figures 3 and 4 show that although Bengkulu has the highest ratio of physicians to population in the southern half of Sumatra Island, within the province the distribution of physicians is only concentrated in urban areas, most of them in Kota Bengkulu, the capital of the province. Users can further drill down to the lowest level of sub-district. Figure 5 below shows the ratio of physicians/100,000 populations in the sub-district level within Bengkulu Utara county.

### Case Study 2: The malaria mortality cases in rural and urban areas

The mortality rate due to malaria is usually higher in rural areas. Using the multidimensional analytical feature in SOVAT, users can determine if this is the case with Indonesia. To do this analysis, "Mortality Number" is used as a measurement in conjunction with a dimension called "Diseases." Malaria is one of the diseases being tracked in the "Disease" dimension. In addition to the "Mortality Number" measurement and "Diseases" dimension, we also use another dimension called "Area", whose members comprise "Urban" and "Rural." By analyzing the data using two dimensions (disease and urban-rural areas), we can compare the mortality number of malaria in urban and rural areas.

**Figure 6:**
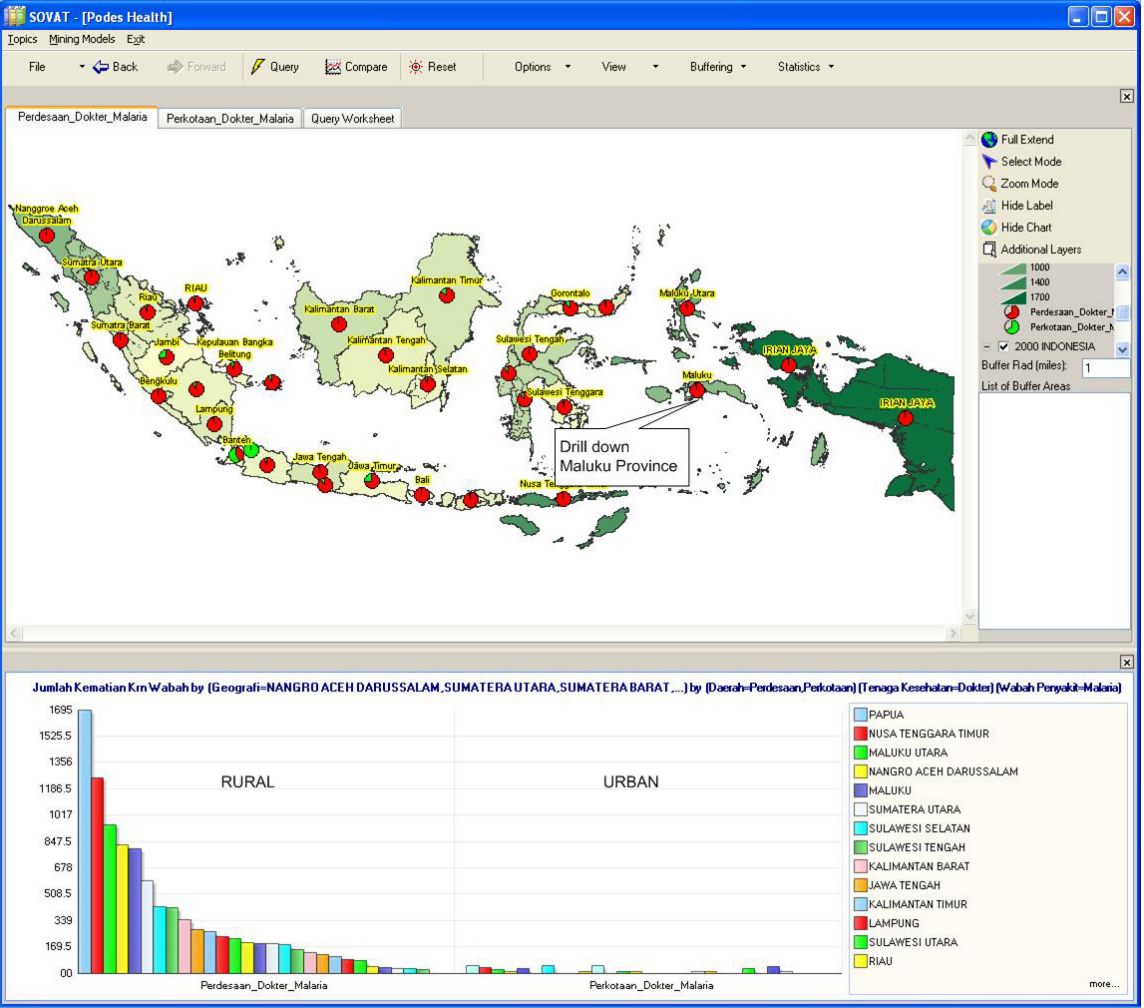
Malaria Mortality Cases in Rural and Urban Areas in Indonesia.

Figure 6 shows that the mortality number of malaria is concentrated in the eastern remote islands of Indonesia (the area with the darkest color). The pie chart inside the map shows the comparison of malaria cases between urban and rural area. The green color represents cases in urban area, while the red one represents cases in rural area. The pie chart shows that malaria mortality is highest in rural areas. The province of Maluku, which is located next to Papua in the eastern part of Indonesia, has the second highest malaria mortality rate. Users might want to focus on Maluku province by clicking at Maluku province and selecting the drill-down menu.

**Figure 7:**
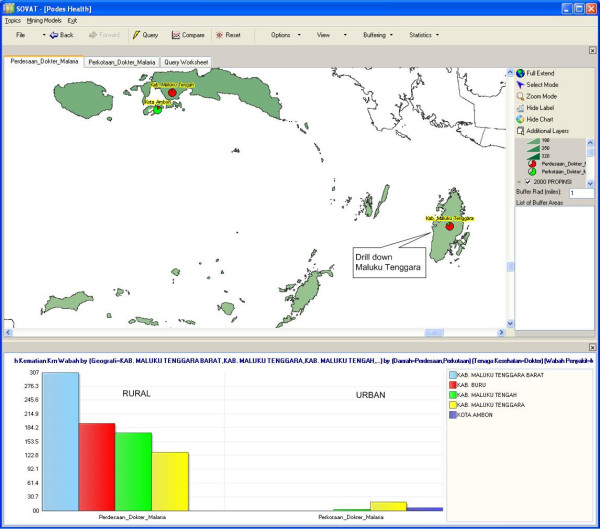
**Malaria Mortality Cases in Rural and Urban Areas in Maluku.** (Second highest).

**Figure 8:**
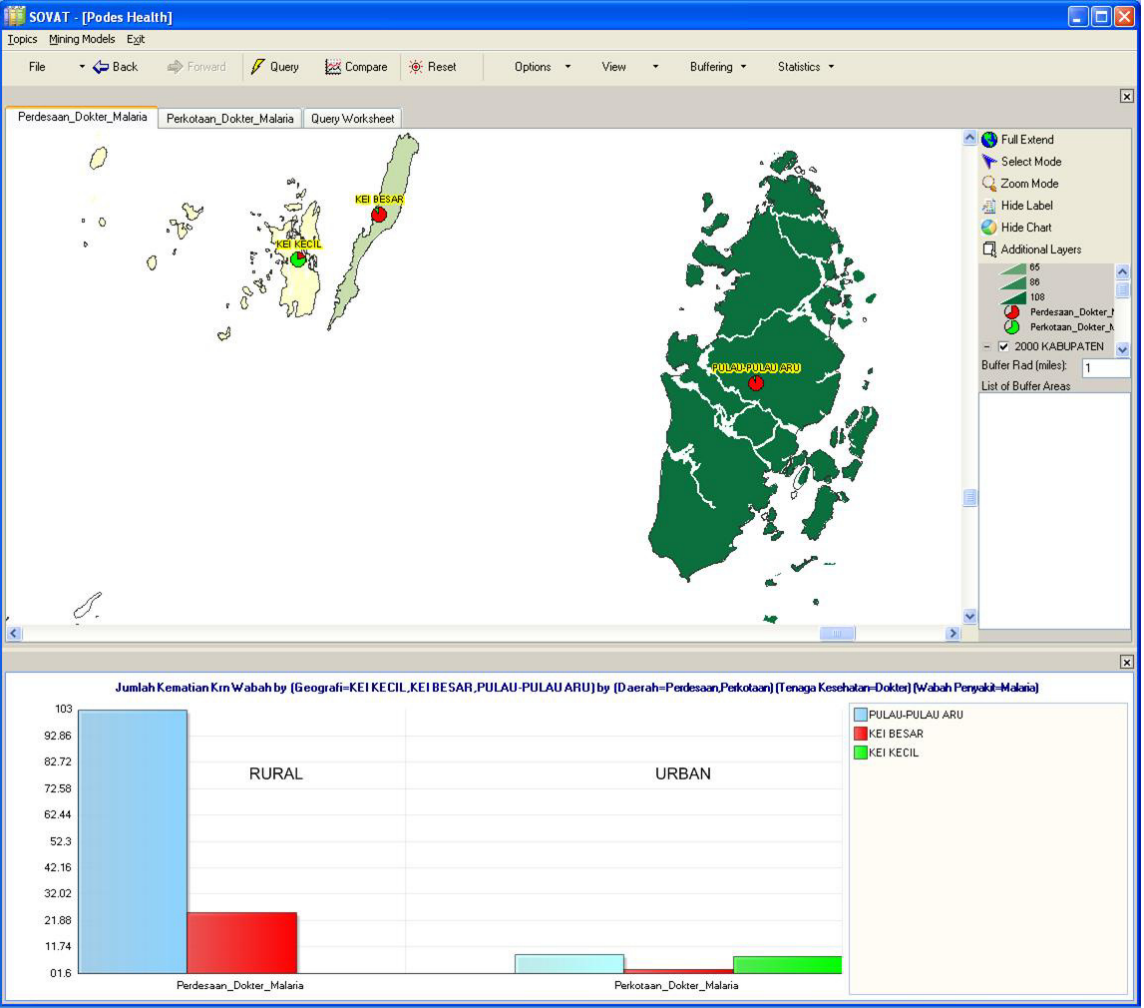
Malaria Mortality Cases in Rural and Urban Areas in South-Eastern Maluku Regency.

The result, as shown in Figure 7, shows that the highest malaria cases is Maluku Tenggara (Southeast Maluku) county. To get a closer look at that county, we can drill down to the lowest level as shown in Figure 8. The comparison shows malaria cases within Southeast Maluku county, for both rural and urban areas.

### Regency

This kind of analysis allows public health professionals to determine which areas need priority in malaria prevention. Our results conclude that the eastern part of Indonesia, especially the rural areas, is in greatest need.

## Conclusion

The case studies show the potential of spatial and multidimensional analysis using SOVAT for CHA in developing countries.

In CHA, timely information is important for decision making. The process of data integration and analyses has been the stumbling block in the CHA process. Using an integrated tool that makes the process fast and easy will allow the assessment to be conducted rapidly. A faster CHA process, in turn, will provide timely information to support the decision-making process by public health professionals.

The strength of multidimensional and spatial analysis is that it allows the user to see the information visually on a map that is otherwise hidden in the complexity of the data and variables. Since the information is presented visually and no statistical skills are required, this type of decision-support system can potentially be used by more users and higher level executives.

Spatial and multidimensional analysis can be even more useful when extensive data from various sources are available. The case studies presented in this paper can be extended by adding more data sets, for example disease surveillance data, hospitalization data, or environmental data. The more data sets integrated into the decision-support system, the more dimensions and richer information that can potentially be uncovered by the system, hence providing higher quality of information for public health decision makers.

SOVAT is an integrated decision support system, and is not designed to replace GIS systems such as ArcGIS. The map capabilities in SOVAT include basic operations such as zoom in/out and buffering, handling of vector data such as point and line, exporting to an image, and printing the map. However, the map in the current version of SOVAT cannot be used to handle more advanced GIS operations such as map projection and map customization.

## Methods

### Spatial and Multidimensional Visualization using SOVAT

SOVAT (Spatial OLAP Visualization and Analytical Tool) is a novel decision-support system developed by the University of Pittsburgh [4]. SOVAT combines two key technologies: On-Line Analytical Processing (OLAP) and a Geographic Information System (GIS) to provide advanced visualization and analyses for large multidimensional data sets. OLAP technology supports multidimensional data modeling that allows for rapid queries of multidimensional data and enables powerful analysis and discovery through a visual display on easy-to-use graphical user interfaces. With OLAP, data are represented conceptually as a multidimensional cube which enables the user to view different dimensions of multiple datasets and then query several dimensions at once. OLAP supports several distinct functions for data retrieval and analysis, such as: drill-up (decreasing granularity, for example, from data by country to data by province), drill-down (increasing granularity, for example, from province to country), and slice and dice (retrieving a sub-section of data, for example, data for May and June for only one province). All of these functions act on the multidimensional data cube and are performed almost instantaneously.

**Figure 9:**
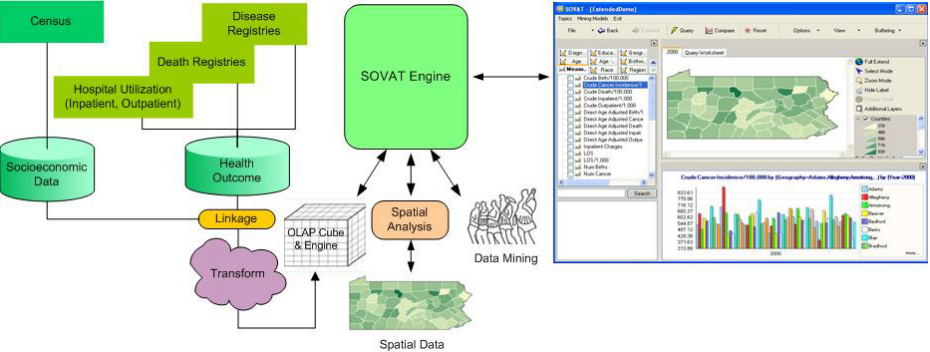
SOVAT Architecture.

The architecture of SOVAT is shown in Figure 9. SOVAT architecture consists of a back-end engine and a front-end application. The back-end engine performs data warehouse (OLAP) functions to process the numerical data and GIS functions to do spatial analysis. An advanced integration module in the back-end engine combines the outcome of OLAP and GIS functions. SOVAT is also equipped with an advanced linkage module capable of integrating an array of health-related databases (inpatient and outpatient hospitalizations; cancer, birth and death registries) and socio-economic data sets. Finally, a front end application fetches outcomes of the integration module and visualizes the outcomes to end users. The current version of SOVAT is a desktop application that runs either on a stand-alone PC or on a client-server environment (SOVAT interface runs on the desktop and the database engine runs on the server, hence the terms "front-end applications" and "back-end engine"). In its current version, SOVAT is not designed to run over the Internet.

**Figure 10:**
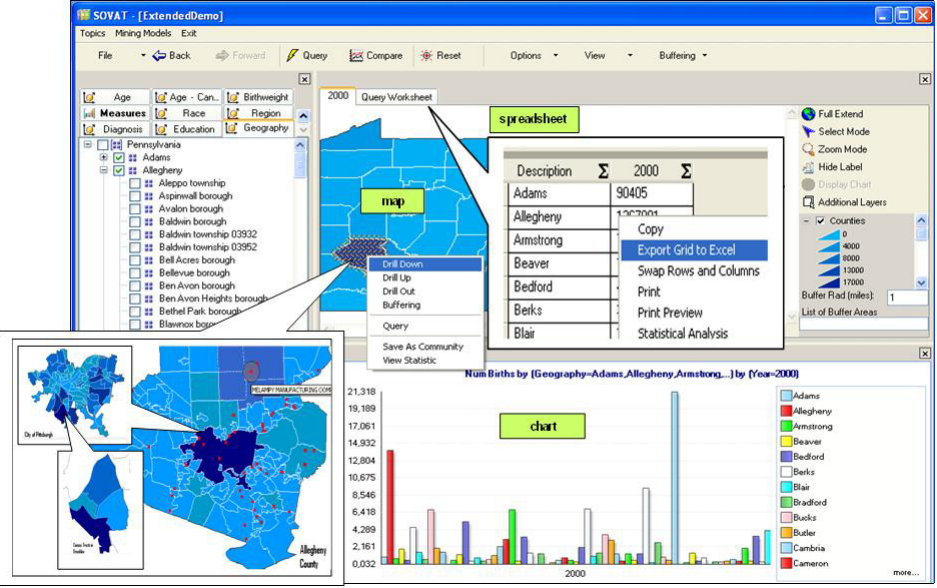
**SOVAT Interface**.

Through an easy-to-use point-and-click interface, even a novice user is able to conduct complex queries quickly and effortlessly using SOVAT. SOVAT DSS has been designed to present health information in a way that will facilitate planning efforts among community stakeholders with diverse interests. The two main components that make up SOVAT interface are the navigation and visualization component [7], as displayed in Figure 10. Navigation components (on the left side) display cube dimensions and their members as a hierarchical tree. These trees provide simplicity for users to browse and select dimension members to be included in their queries. A search engine is provided as a complementary feature to help users find a member in a dimension with large members. The visualization component (on the right side) consists of spreadsheets, charts, and maps. SOVAT provides a spreadsheet to display query results, translates the spreadsheet data into charts, and visualizes the results onto maps. Several advantages of having different approaches include the ability to easily recognize certain trends using charts and the ability to recognize geographic patterns using the maps. The map in SOVAT is a real vector GIS (not a bitmap image) that can be used to query an area by clicking the map, drill down to lower geographic levels, drill up to upper levels, and querying neighbourhood areas. SOVAT is also equipped with the ability to export query results to be used in different applications. For example, users can export the spreadsheets into Excel format and save the grid/map as an image to be displayed in other applications. In addition to these functionalities, the queries can also be saved to be used at a later time.

### Data Sets

The Indonesian data sets are comprised of demographic data, health indicators, and spatial data (maps). The data sets come in different levels of detail and were collected using different collection methods. Indonesia is divided into provinces. Provinces consist of regencies (kabupaten) and cities (kota) which together are called counties in this paper. One level below counties is sub-district (kecamatan), while the lowest administrative level – that is the one below sub-district – is called village (desa). The definition of village applies to both rural and urban areas. Political changes in Indonesia since the last decade have affected the administrative division, with the tendency of a growing number of provinces and regencies. The latest data from the Ministry of Internal Affairs show that Indonesia currently has 33 provinces and 445 counties [8]. There are more than 75 new counties since the year 2000, an increase of more than 20%.

### Statistical Data: Census and Village Statistics

Data sets from the Indonesian Census and the Indonesian village statistics were used in the case studies. Similar to many countries, a census in Indonesia is conducted every decade. Indonesia conducts a series of population, agricultural, and economic censuses. The population census takes place in the years ending with "0"; the agricultural is conducted in the years ending with "3"; while the economic census is held in the years ending in "6" [9]. Of these censuses, the population census is the most comprehensive and is aimed at gathering characteristics of the Indonesian population such as gender, age, marital status, education level, and occupation. The 2000 Population Census is the latest census and the first census conducted using complete enumeration. Since the 2000 Census was aimed at providing users with small area statistics, statistics of villages can be established from the data collected. In addition to the censuses, the Indonesian Bureau of Statistics also conducts an intercensal population survey (SUPAS) in between the two censuses [10]. The survey is designed to collect the population statistic that is comparable to the population census. Another approach to data collection, rather than to collect data on each household and individual, is to collect statistics on villages [11,12]. Village statistics, called Potensi Desa (PODES), are the main data source of this project. Village statistics provide information that otherwise is not available. Among the objectives of village-level data collection are:

• Providing information of potential and actual development in the village by providing socio-economic conditions and available facilities;

• Providing a database for regional planning as well as a progress report on the development at the village-level; and,

• Providing core data of the small area statistics.

The information in village-level collection includes: the number of the population and households, the housing and environmental data, the education and health-related data, socio-cultural information, recreation and sport facilities, transportation, and communication.

While demographic data are mostly from the Bureau of Statistics (BPS), more specific health indicators are available from the Ministry of Health [13]. These data include: a general mortality rate, an infant mortality rate, life expectancy, top diagnoses for in-patients and out-patients, and morbidity of infectious diseases Although these data are not used for this project, it is a potential source to use in future works.

### Spatial Data

SOVAT uses spatial data in polygon format that consists of administrative-boundary maps. The map is rendered using certain color schemes to display the results of OLAP queries. For example, in Figure 2, the darker the color, the higher are the results of performed queries. In addition to the polygon data, SOVAT can also have additional layers using lines and point data, for example to represent rivers, streets, cities, or industrial places. The additional layers can be used to perform other spatial analysis such as buffering.

The digital map of Indonesia is provided by the BPS. The existing spatial data come from four different levels: from province level down to village level. However, due to the low accuracy of the village-level spatial data, we chose to use one level higher than the village – that is the sub-district (kecamatan) level.

### Data Linkage and Multidimensional Modeling

Geographic location was used as the primary linkage variable that connects statistical and spatial data sets. The linking process is done using an administrative code that is uniquely defined for every administrative unit. Standardization is the key for the linking process. Most developed countries have a uniform identification for every geographic entity that can be used by the government and private sector. For example, in the United States there is the Federal Information Processing Standards codes (FIPS codes), a standardized code for every geographic entity in the US issued by the National Institute of Standards and Technology (NIST). This code is used by the US Census Bureau and other government agencies that generate statistical data sets.

Unfortunately, there is no such uniform identification standard for geographic entities in Indonesia. The lack of a uniform code leads to the use of geographic names (such as the name of counties and villages) as the key identifiers of the geographic entity. Geographic names are very susceptible to typographical errors and inconsistent spelling. As a result, the same geographic entities can be written differently in different reports even if the reports come from the same government institution (Bureau of Statistics). Several solutions were tried for this problem. Some of the data were corrected using a pattern-matching approach, while the remainder that could not be recognized using pattern matching were manually corrected.

The need of a uniform code is more important in light of the rapid changes of administrative boundaries in the past decade. The problem with spatial data becomes more complex since the updating process of spatial data is not as fast as the process of administrative changing. While the administrative code is easily updated with more recent changes, the map is still outdated. For example, the administrative boundaries are changed up to 2007, however the latest version of the map we use is from 2000.

Since there is no official new map released, there is no other option rather than to translate the data into an older map.

**Figure 11:**
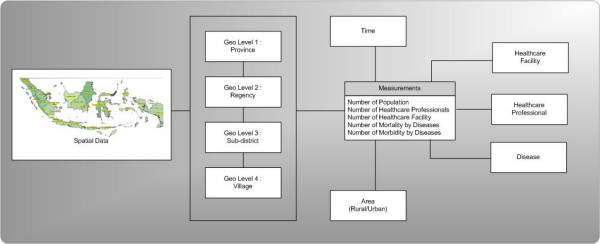
Linkage and dimensional modelling.

As shown in Figure 11, the geographic unit provides a linkage between spatial data and other numerical data sets. Multidimensional database design was conducted in order to develop the database capable of supporting multidimensional analysis. The "measures" in this model is a statistical number about the geographic unit such as the number of population in a sub-district. The "dimension" is an independent variable that allows us to view the information from different angles. For example, we can view the number of incidences based on the disease type, urban-rural designation area, or time. We can also simultaneously "slice" and "dice" the information using all available dimensions (for example, the number of malaria incidences in a rural area in the province of Papua in the year 2000).

## Competing interests

The authors declare that they have no competing interests.

## Authors' contributions

BP and MVP conceived the system, conducted the analyses, and wrote the final version of the manuscript. WS and MVP conducted data integrations. GP wrote the program for and conducted data cleansing. MS wrote the abstract and involved in the development of SOVAT, DSB lead the MIDAS Indonesian project that initiated the collection of Indonesian health statistics.

## References

[B1] Committee for the Study of the Future of Public Health, Division of Health Care Services, Institute of Medicine (1988). The Future of Public Health.

[B2] Bedard Y, Gosselin P, Jerrett M, Elliott SJ, Catelan R, Poitras P, Gingras A (2000). GIS and OLAP in Health Surveillance: Needs Analysis for Successful Integration.

[B3] Scotch M, Parmanto B, Gadd C, Sharma R (2006). Exploring the role of GIS during community health assessment problem solving: experiences of public health professionals. International Journal of Health Geographics.

[B4] Scotch M, Parmanto B (2006). Development of SOVAT: A numerical-spatial decision support system for community health assessment research. International Journal of Medical Informatics.

[B5] United Nations Environment Programme (UNEP) National Rapid Environmental Assessment – Indonesia.

[B6] Officer for the Coordination of Humanitarian Affairs Map of Poverty and Health Facility Damaged.

[B7] Scotch M, Parmanto B, Monaco V (2007). Usability Evaluation of the Spatial OLAP Visualization and Analysis Tool (SOVAT). Journal of Usability Studies.

[B8] Indonesia Ministry of Internal Affairs (2004). Code and Data Administrative Areas in Indonesia.

[B9] Dwijosumono S (2001). National Statistical System: A statistical strategies in Indonesia. BPS-Statistics, Jakarta Indonesia.

[B10] Data Statistik Indonesia (Statistics Indonesia).

[B11] Smeru Research Report Developing a Poverty Map for Indonesia: A Tool for Better Targeting in Poverty Reduction and Social Protection Programs.

[B12] Food and Agriculture Organization of United Nations Collection of Village-Level Data Through The 2003 Agriculture Census and The 2006 Economic Census in Indonesia.

[B13] Bank Data Department Kesehatan RI (Indonesia Ministry of Health Data Repository).

